# Immunopharmacological considerations of general anaesthetics for surgical procedures in the times of COVID-19: Correspondence

**DOI:** 10.1097/MS9.0000000000000555

**Published:** 2023-04-06

**Authors:** Firzan Nainu, Andri Frediansyah, Emil Salim, Deepak Chandran, Kuldeep Dhama, Ali A. Rabaan, Harapan Harapan, Talha Bin Emran

**Affiliations:** a Department of Pharmacy, Faculty of Pharmacy, Hasanuddin University, Makassar; b PRTPP, National Research and Innovation Agency (BRIN), Yogyakarta; c Department of Pharmacology, Faculty of Pharmacy, Universitas Sumatera Utara, Medan; d Medical Research Unit; e Department of Microbiology; f Tropical Disease Centre, School of Medicine, Universitas Syiah Kuala, Banda Aceh, Indonesia; g Department of Veterinary Sciences and Animal Husbandry, Amrita School of Agricultural Sciences, Amrita Vishwa Vidyapeetham University, Coimbatore; h Division of Pathology, ICAR-Indian Veterinary Research Institute, Izatnagar, Bareilly, Uttar Pradesh, India; i Molecular Diagnostic Laboratory, Johns Hopkins Aramco Healthcare, Dhahran; j College of Medicine, Alfaisal University, Riyadh, Saudi Arabia; k Department of Public Health and Nutrition, The University of Haripur, Haripur, Pakistan; l Department of Pharmacy, BGC Trust University Bangladesh, Chittagong; m Department of Pharmacy, Faculty of Allied Health Sciences, Daffodil International University, Dhaka, Bangladesh

*Dear Editor*,

Since its discovery in December 2019, the novel severe acute respiratory syndrome coronavirus 2 (SARS-CoV-2) has caused more than 6.88 million deaths and more than 676 million cases of coronavirus disease 2019 (COVID-19) worldwide as of 10 March 2023^1^. Face masks, hand washing, proper sanitation, contact tracing, lockdowns, early confirmatory testing, and fortified medical facilities have all helped to curb the rapid spread of SARS-CoV-2^2^. Despite a worldwide vaccination drive meant to protect communities and promote herd immunity, the virus has not been contained yet^3^,^4^. This is mostly attributed to the recurring introduction of novel variants and mutations of SARS-CoV-2, which has caused multiple waves, and the current rapid increase in COVID-19 cases, which represents the fourth wave and is primarily attributable to the recently developed Omicron variant^2^.

Recent global healthcare systems have been severely impacted by the emergence of COVID-19^5^,^6^. The variety of disease severity, prolonged infectious periods, and common clinical symptoms and causation contribute to the difficulty of diagnosis, treatment, and resource allocation^7^. Furthermore, COVID-19 outbreaks have led to significant restrictions on surgical practice and education^8^. In the 12 weeks following the first pandemic surge, more than 28 million surgical procedures were cancelled due to COVID-19 infections^9^, with millions of people awaiting surgical treatment^10^. There is new study that indicates an increased risk of mortality for surgical patients who become infected with COVID-19 within the first six weeks after their operation^10^. The American Society of Anaesthesiologists and the Anaesthesia Patient Safety Foundation have come together to issue a joint recommendation based on the data that is currently available. This recommendation is to delay surgery for 4–12weeks after a diagnosis has been made, with the variable timeline depending on the severity of COVID-19 and vaccination status^11^,^12^.

COVID-19 primarily not only affects the pulmonary system, but it is also known to cause endothelial dysfunction^13^, vascular inflammation^14^, and sweeping changes in the coagulation cascade^15^. It is believed that all these things contribute to an increase in the risk of developing deep vein thrombosis^16^, pulmonary embolus^17^, and cerebrovascular accident^18^–^20^. Moreover, pulmonary problems, cardiac injury, and acute renal injury have been related with cytokine release and localized inflammation^21^,^22^. Collectively, these relationships create a substantial potential mortality risk for postoperative patients who have higher inflammatory activation due to surgery. This risk may raise the morbidity and mortality rate among surgery patients. There is evidence that suggests a 30-day mortality rate of 19.1% in elective surgical patients and a 30-day mortality rate of 26.0% in emergency surgical patients^9^. Furthermore, approximately half of patients who underwent surgery while infected with SARS-CoV-2 experienced postoperative pulmonary complications^9^. In addition, given the scope of the pandemic, perioperative outcomes following a previous SARS-CoV-2 infection are a major concern, as a considerable number of patients who have been previously infected would require surgery^10^. Infection with SARS-CoV-2 during surgery is associated with a 10-fold increase in short-term mortality^23^. Therefore, it is crucial to minimize the danger of patients entering the hospital while incubating SARS-CoV-2 or contracting the virus in the hospital. This is especially critical for individuals at high risk of severe disease and mortality from COVID-19 including comorbidities. Concurrently, emerging SARS-CoV-2 mutations and subvariants have resulted in the reinstatement of hygienic measures and limitations in several countries all over the world^24^.

In addition, having surgery during or close to the time of an active SARS-CoV-2 infection is linked to an increased risk of developing postoperative complications, such as postoperative pneumonia^25^, respiratory failure^26^, pulmonary embolism^17^, and sepsis^27^, arrhythmia^28^, renal failure^29^, urinary tract infection^30^,^31^, and deep vein thrombosis^32^. Surgery conducted between 4 and 8 weeks after a confirmed case of SARS-CoV-2 infection is still associated with an increased risk of the patient acquiring postoperative pneumonia^30^. However, there is no increased risk of developing postoperative problems linked with surgery carried out 8 weeks or later after a confirmed SARS-CoV-2 infection^9^. After a waiting period of 8 weeks from the initial date of confirmed SARS-Cov-2 infection, the patient could have surgery (or the time that likely has less SARS-CoV-2 antibodies in the blood).

Several pharmaceutical preparations are subject for use during surgical procedures. One of those is general anaesthetics. General anaesthetics is a reversible drug-induced sleep-like state, that can generate unconsciousness, amnesia, antinociception, and immobility of the patients, while maintaining their physiological stability^33^. The typically employed general anaesthesia are intravenous and inhaled drugs^34^. The most often used intravenous anaesthetics include propofol, thiopental, ketamine, and etomidate^35^ which induce anaesthesia by increasing the efficacy of signalling through the inhibitory gamma-aminobutyric acid A (GABA_A_) receptors^36^.

Propofol, a strong intravenous hypnotic/sedative drug, is used as a first-line medicine for the sedation of critically ill patients undergoing intensive care, permitting potentially life-saving invasive interventions such as mechanical ventilation^37^. It exhibits rapid and smooth induction with almost no excitation phenomena, a relatively short context-sensitive time, a rapid terminal half-life, and a low incidence of postoperative nausea and vomiting, making it a highly versatile hypnotic agent. It is utilized for sedation and anaesthesia for nearly all types of surgery but is particularly well-suited for anaesthesia in patients having ambulatory surgery^38^ and neurosurgery, where rapid psychomotor recovery is of paramount importance. Its efficacy and utility have also been proven for the sedation of patients in the intensive care unit^39^ and conscious sedation of patients undergoing diagnostic or invasive procedures^40^.

Thiopental, the most commonly used barbiturate^35^, is another intravenous anaesthetic administered to intensive care patients, typically for treating patients with refractory status epilepticus or intracranial hypertension^37^. Another intravenous anaesthetic is ketamine hydrochloride, a nonbarbiturate dissociative anaesthetic that swiftly acts and generates profound anaesthesia and analgesia^41^–^43^. Ketamine is an excellent medication for short-term medical treatments that do not require the relaxation of skeletal muscles. It is used for general anaesthesia induction as a pre-anaesthetic to other general anaesthetic drugs^44^–^46^. Etomidate, a nonbarbiturate hypnotic with a very short half-life, is commonly used to induce anaesthesia in intensive care unit patients due to its acceptable hemodynamic profile and quick onset. The advantage of etomidate is that it reduces the risk of induction hypotension, which can lead to coronary hypoperfusion, dysrhythmia, and cardiac arrest. Etomidate, on the other hand, lowers adrenocortical activity by inhibiting 11-hydroxylase^47^. Even though etomidate has some adverse side effects (such as adrenal cortex inhibition, hiccup, myoclonus, injection pain, nausea, and vomiting), it is still a good choice for critically ill patients because of its positive properties (such as its rapid onset, short duration, stable cardiovascular profile, minimal respiratory depression, improved endotracheal intubation conditions, lack of histamine release, and rarity of allergic reactions)^48^–^52^.

Inhaled anaesthetics or volatile anaesthetics can be used to administer general anaesthesia to a patient undergoing surgery^53^,^54^. In addition, volatile anaesthetics produce their effects rapidly. The majority of individuals who are sedated by these medicines recover swiftly and easily. Using contemporary equipment, their concentrations can be precisely checked, and their application is quite simple. In clinical practice, volatile anaesthetics have been widely utilized for these reasons^54^. In the United States, modern volatile anaesthetics include isoflurane, sevoflurane, and desflurane^54^. Halothane was used in clinical settings for more than 40 years, but it was replaced by newer volatile anaesthetics in the 1990s^55^. Inhalation anaesthetics are used to initiate and maintain general anaesthesia in the operating room. The volatile anaesthetics (halothane, isoflurane, desflurane, and sevoflurane) are liquids at room temperature and must be administered using vaporizers. All inhalational anaesthetics cause amnesia and immobility, except for nitrous oxide, which also relieves pain. Inhaled anaesthetics are often used along with intravenous anaesthetics. In the intensive care unit, inhaled anaesthetics are mostly used for sedation, refractory bronchospasm, and control of status epilepticus that is unresponsive to anticonvulsant medicines^56^. To execute anaesthetic effects, volatile anaesthetics target certain central nervous system receptors, such as the neuronal GABA_A_ receptor, N-methyl-D-aspartate (NMDA) receptor, and glutamate receptor subtypes^57^.

Anaesthetic drugs have been reported able to modify immune function, which has the potential for considerable implications on perioperative infections and surgical outcomes^58^. Several studies have shown the immunosuppressive effect of anaesthetic drugs. For instance, propofol has a mixed effect on innate immune system cells, particularly macrophages and natural killer (NK) cells. Propofol inhibits mouse macrophage chemotaxis and oxidative capacity, as well as the synthesis of ATP and interferon-γ^59^. There is evidence that it may be less toxic to NK cells, which is significant for cancer patients undergoing anaesthesia because NK cells are known to have antiviral and anticancer properties^60^,^61^. In a rat model, unlike halothane and ketamine, propofol did not inhibit NK cells or promote metastases^60^. Further research on a human cell model revealed that propofol does not reduce the cytotoxicity caused by NK cells (like sevoflurane and isoflurane did by inhibiting LFA-1)^61^. This indicates that while propofol suppresses macrophage activity, its influence on NK cells may be advantageous in the perioperative treatment of viral and neoplastic conditions.

Another general anaesthetics, etomidate, can suppress cell-mediated innate immunity by inhibiting the chemotaxis of eosinophils *in vitro* 62. Etomidate may potentially have a negative impact on the adaptive immune system. In rats with sepsis treated with etomidate, there was an increase in lymphocyte apoptosis^63^. In addition, in-vitro measurements of phytohaemagglutinin-stimulated lymphocyte proliferation revealed that typical clinical plasma concentrations of etomidate depressed total T-lymphocyte populations^64^. Ketamine possesses anti-inflammatory and immunosuppressive effects, which are mediated by the innate and adaptive immune systems. In rats and humans, ketamine inhibited neutrophil chemotaxis by decreasing the expression of endothelium adhesion molecules. The generation of superoxide free-radical was blocked through decreased phosphorylation of the NADPH oxidase enzyme complex^65^. Ketamine was found to inhibit the differentiation of monocytes into immature dendritic cells (DCs) via an NMDA-dependent and transforming growth factor beta (TGF)-dependent mechanism. DCs are members of the innate immune system and play a critical role in antigen presentation for adaptive T-cell function^65^. At therapeutically relevant doses, dexmedetomidine had no effect on neutrophil chemotaxis, phagocytosis, or superoxide generation^66^.

The majority of volatile anaesthetics bind to the receptors located in the central nervous system (“canonical targets”) in order to generate anaesthesia. These receptors include γ-aminobutyric acid type A (GABAA) receptor, NMDA receptor and two-pore-domain K+ (K2P) channel^67^. In addition to canonical target molecules, volatile anaesthetics also target TLR2, TLR4, LFA-1, Mac-1, and Rap1 (“non-canonical targets”) on immune cells, which may suppress the functions of neutrophils, monocytes, and macrophages^68^. Neutrophils and macrophages both generate a variety of cytokines. A broad range of neutrophil receptors, including GPCRs, FcRs, Mac-1, and TLRs, induce cytokine production^69^. Among them, Mac-1, TLR2, and TLR4 are targets of volatile anaesthetics^68^. Volatile anaesthetics influence blood cytokine levels and reduce proinflammatory cytokine concentrations^70^. Extended isoflurane exposure was related to poor neutrophil recruitment and bacterial phagocytosis via the reduced activity of leucocyte function-associated antigen-1 (LFA-1) and macrophage-1 antigen (Mac-1), which are important adhesion molecules on leucocytes^71^. The usage of volatile anaesthetics has been directly connected to decreased phagocytic activity and chemotaxis. For instance, isoflurane decreases the ability of phagocytes to ingest opsonized and non-opsonized particles in a time-dependent manner^72^. Via dose-dependent actions, sevoflurane and desflurane promote apoptosis of thymus T cells, and desflurane induces apoptosis of B lymphocytes by activating IP3 (inositol triphosphate)^67^,^72^, which may contribute to immunological suppression after surgical procedures.

Opioids, with morphine being the most thoroughly researched, can yield immunosuppressive effects. Opioids inhibit NK cell cytotoxicity, reduce macrophage-mediated and neutrophil-mediated phagocytosis, inhibit the generation of reactive oxygen species by neutrophil, and downregulate cytokine synthesis^73^,^74^. Additional opioids-induced phenotypical characteristics such as less production of chemokine by neutrophils, reduced antigen presentation by DCs, and increased permeability of the intestinal barrier have also been reported^73^,^75^. Regarding the adaptive immunity, it has been demonstrated that opioids induce apoptosis, impede immune cell proliferation, and inhibit T-cell-mediated adaptive responses^73^,^74^. Moreover, B lymphocyte effector response is diminished and Th1 cell death and Th2 differentiation are increased, which further impaired host ability in the pathogen clearance^73^,^75^. Overall, the inhibition of the immune system by anaesthetic medications may facilitate perioperative infections and may significantly affect post-surgical outcomes.

In conclusion, relevant evidence indicated that certain general anaesthetics may exert suppressive effects on the innate and adaptive immune responses, leaving patients vulnerable to SARS-CoV-2 infection during surgery. In light of such evidence, highest level of precaution shall be taken to prevent exacerbations of infection during and after the application of general anaesthetics. This is important to achieve the best outcome in the medical-surgical interventions amid the absence of alternative medications with better pharmacological properties but less immunosuppressive side effects.

## Ethical approval

The authors declare no involvement of animal studies or human participants in the study as it is a correspondence article.

## Source of funding

This research did not receive any specific grant from funding agencies.

## Author contribution

F.N., A.F., and E.S. designed the study; F.N., A.F., and E.S. wrote the first draft; F.N., A.F., E.S., D.C., K.D., A.A.R., H.H., and T.B.E. revised the draft; F F.N., A.F., E.S., D.C., K.D., A.A.R., H.H., and T.B.E. reviewed the final draft. All authors have critically reviewed and approved the final draft and are responsible for the content and similarity index of the manuscript.

## Conflicts of interest disclosure

All authors declare that there is no exist of commercial or financial relationship that could, in any way, lead to a potential conflict of interest.

## References

[1] Dong E Du H Gardner L . An interactive web-based dashboard to track COVID-19 in real time. Lancet Infect Dis 2020;20:533–534. 32087114 10.1016/S1473-3099(20)30120-1 PMC7159018

[2] Dhama K Nainu F Frediansyah A . Global emerging Omicron variant of SARS-CoV-2: Impacts, challenges and strategies. J Infect Public Health 2023;16:4–14. 36446204 10.1016/j.jiph.2022.11.024 PMC9675435

[3] Watson OJ Barnsley G Toor J . Global impact of the first year of COVID-19 vaccination: a mathematical modelling study. Lancet Infect Dis 2022;22:1293–1302. 35753318 10.1016/S1473-3099(22)00320-6 PMC9225255

[4] Lazarus JV Wyka K White TM . A survey of COVID-19 vaccine acceptance across 23 countries in 2022. Nat Med 2023;29:366–375. 36624316 10.1038/s41591-022-02185-4

[5] Haldane V De Foo C Abdalla SM . Health systems resilience in managing the COVID-19 pandemic: lessons from 28 countries. Nat Med 2021;27:964–980. 34002090 10.1038/s41591-021-01381-y

[6] Myers LC Liu VX . The COVID-19 pandemic strikes again and again and again. JAMA Network Open 2022;5:e221760. 35262720 10.1001/jamanetworkopen.2022.1760

[7] Dhama K Khan S Tiwari R . Coronavirus disease 2019-COVID-19. Clin Microbiol Rev 2020;33:e00028–20. 32580969 10.1128/CMR.00028-20 PMC7405836

[8] Sohrabi C Mathew G Franchi T . Impact of the coronavirus (COVID-19) pandemic on scientific research and implications for clinical academic training—a review. Int J Surg 2021;86:57–63. 33444873 10.1016/j.ijsu.2020.12.008 PMC7833269

[9] Collaborative C . Elective surgery cancellations due to the COVID-19 pandemic: global predictive modelling to inform surgical recovery plans. Br J Surg 2020;107:1440–1449. 32395848 10.1002/bjs.11746 PMC7272903

[10] El-Boghdadly K Cook TM Goodacre T . SARS-CoV-2 infection, COVID-19 and timing of elective surgery: a multidisciplinary consensus statement on behalf of the Association of Anaesthetists, the Centre for Peri-operative Care, the Federation of Surgical Specialty Associations, the Royal College of Anaesthetists and the Royal College of Surgeons of England. Anaesthesia 2021;76:940–946. 33735942 10.1111/anae.15464 PMC8250763

[11] Barie PS Brindle ME Khadaroo RG . Omicron, long-COVID, and the safety of elective surgery for adults and children: Joint Guidance from the Therapeutics and Guidelines Committee of the Surgical Infection Society and the Surgery Strategic Clinical Network, Alberta Health Services. Surg Infect (Larchmt) 2023;24:6–18. 36580648 10.1089/sur.2022.274

[12] Bryant JM Boncyk CS Rengel KF . Association of time to surgery after COVID-19 infection with risk of postoperative cardiovascular morbidity. JAMA Network Open 2022;5:e2246922. 36515945 10.1001/jamanetworkopen.2022.46922 PMC9856239

[13] Bonaventura A Vecchié A Dagna L . Endothelial dysfunction and immunothrombosis as key pathogenic mechanisms in COVID-19. Nat Rev Immunol 2021;21:319–329. 33824483 10.1038/s41577-021-00536-9 PMC8023349

[14] Zhang J Tecson KM McCullough PA . Endothelial dysfunction contributes to COVID-19-associated vascular inflammation and coagulopathy. Rev Cardiovasc Med 2020;21:315–319. 33070537 10.31083/j.rcm.2020.03.126

[15] Song WC FitzGerald GA . COVID-19, microangiopathy, hemostatic activation, and complement. J Clin Invest 2020;130:3950–3953. 32459663 10.1172/JCI140183 PMC7410042

[16] Zhang L Feng X Zhang D . Deep vein thrombosis in hospitalized patients with COVID-19 in Wuhan, China: prevalence, risk factors, and outcome. Circulation 2020;142:114–128. 32421381 10.1161/CIRCULATIONAHA.120.046702

[17] Poissy J Goutay J Caplan M . Pulmonary embolism in patients with COVID-19: awareness of an increased prevalence. Circulation 2020;142:184–186. 32330083 10.1161/CIRCULATIONAHA.120.047430

[18] Avula A Nalleballe K Narula N . COVID-19 presenting as stroke. Brain Behav Immun 2020;87:115–119. 32360439 10.1016/j.bbi.2020.04.077 PMC7187846

[19] Parsay S Vosoughi A Khabbaz A . The incidence and mortality ratio of ischemic cerebrovascular accidents in COVID-19 cases: a systematic review and meta-analysis. J Stroke Cerebrovasc Dis 2021;30:105552. 33360509 10.1016/j.jstrokecerebrovasdis.2020.105552 PMC7746086

[20] Syahrul S Maliga HA Ilmawan M . Hemorrhagic and ischemic stroke in patients with coronavirus disease 2019: incidence, risk factors, and pathogenesis - a systematic review and meta-analysis. F1000Res 2021;10:34. 33708378 10.12688/f1000research.42308.1 PMC7934095

[21] Messerer DAC Halbgebauer R Nilsson B . Immunopathophysiology of trauma-related acute kidney injury. Nat Rev Nephrol 2021;17:91–111. 32958893 10.1038/s41581-020-00344-9

[22] Rosner MH Okusa MD . Acute kidney injury associated with cardiac surgery. Clin J Am Soc Nephrol 2006;1:19–32. 17699187 10.2215/CJN.00240605

[23] Abbott TEF Fowler AJ Dobbs TD . Mortality after surgery with SARS-CoV-2 infection in England: a population-wide epidemiological study. Br J Anaesth 2021;127:205–214. 34148733 10.1016/j.bja.2021.05.018 PMC8192173

[24] Yang C . Does hand hygiene reduce SARS-CoV-2 transmission? Graefes Arch Clin Exp Ophthalmol 2020;258:1133–1134. 32221693 10.1007/s00417-020-04652-5 PMC7100395

[25] Nahshon C Bitterman A Haddad R . Hazardous postoperative outcomes of unexpected COVID-19 infected patients: a call for global consideration of sampling all asymptomatic patients before surgical treatment. World J Surg 2020;44:2477–2481. 32418028 10.1007/s00268-020-05575-2 PMC7229873

[26] Li X Ma X . Acute respiratory failure in COVID-19: is it “typical” ARDS? Crit Care 2020;24:198. 32375845 10.1186/s13054-020-02911-9 PMC7202792

[27] Remy KE Brakenridge SC Francois B . Immunotherapies for COVID-19: lessons learned from sepsis. Lancet Respir Med 2020;8:946–949. 32444269 10.1016/S2213-2600(20)30217-4 PMC7195015

[28] Mohammad M Emin M Bhutta A . Cardiac arrhythmias associated with COVID-19 infection: state of the art review. Expert Rev Cardiovasc Ther 2021;19:881–889. 34702128 10.1080/14779072.2021.1997589 PMC8607535

[29] Adapa S Aeddula NR Konala VM . COVID-19 and renal failure: challenges in the delivery of renal replacement therapy. J Clin Med Res 2020;12:276–285. 32489502 10.14740/jocmr4160 PMC7239583

[30] Deng JZ Chan JS Potter AL . The risk of postoperative complications after major elective surgery in active or resolved COVID-19 in the United States. Ann Surg 2022;275:242–246. 34793348 10.1097/SLA.0000000000005308 PMC8745943

[31] Fakih MG Bufalino A Sturm L . Coronavirus disease 2019 (COVID-19) pandemic, central-line-associated bloodstream infection (CLABSI), and catheter-associated urinary tract infection (CAUTI): The urgent need to refocus on hardwiring prevention efforts. Infect Control Hosp Epidemiol 2022;43:26–31. 33602361 10.1017/ice.2021.70 PMC8007950

[32] Suh YJ Hong H Ohana M . Pulmonary embolism and deep vein thrombosis in COVID-19: a systematic review and meta-analysis. Radiology 2021;298:e70–e80. 33320063 10.1148/radiol.2020203557 PMC7745997

[33] Brown EN Pavone KJ Naranjo M . Multimodal general anesthesia: theory and practice. Anesth Analg 2018;127:1246–1258. 30252709 10.1213/ANE.0000000000003668 PMC6203428

[34] Shui M Xue Z Miao X . Intravenous versus inhalational maintenance of anesthesia for quality of recovery in adult patients undergoing non-cardiac surgery: a systematic review with meta-analysis and trial sequential analysis. PLoS One 2021;16:e0254271. 34270584 10.1371/journal.pone.0254271 PMC8284831

[35] Khan KS Hayes I Buggy DJ . Pharmacology of anaesthetic agents I: intravenous anaesthetic agents. Continu Educ Anaesthesia Crit Care Pain 2013;14:100–105.

[36] Canbek O Ipekcioglu D Menges OO . Comparison of propofol, etomidate, and thiopental in anesthesia for electroconvulsive therapy: a randomized, double-blind clinical trial. J ect 2015;31:91–97. 25268043 10.1097/YCT.0000000000000190

[37] Fraser GL Riker RR . Sedation and analgesia in the critically ill adult. Curr Opin Anaesthesiol 2007;20:119–123. 17413394 10.1097/ACO.0b013e32808255b4

[38] Joo HS Perks WJ . Sevoflurane versus propofol for anesthetic induction: a meta-analysis. Anesth Analg 2000;91:213–219. 10866915 10.1097/00000539-200007000-00040

[39] Liu H Ji F Peng K . Sedation after cardiac surgery: is one drug better than another? Anesth Analg 2017;124:1061–1070. 27984229 10.1213/ANE.0000000000001588

[40] Kochhar GS Gill A Vargo JJ . On the horizon: the future of procedural sedation. Gastrointest Endosc Clin N Am 2016;26:577–592. 27372779 10.1016/j.giec.2016.03.002

[41] Nichols KA Paciullo CA . Subdissociative ketamine use in the emergency department. Adv Emerg Nurs J 2019;41:15–22. 30702529 10.1097/TME.0000000000000222

[42] BinKharfi M AlSagre A . BET 2: Safety and efficacy of low-dose ketamine versus opioids for acute pain management in the ED. Emerg Med J 2019;36:128–129. 30696780 10.1136/emermed-2019-208441.2

[43] Kapur A Kapur V . Conscious sedation in dentistry. Ann Maxillofac Surg 2018;8:320–323. 30693254 10.4103/ams.ams_191_18 PMC6327823

[44] Chomchai S Phuditshinnapatra J Mekavuthikul P . Effects of unconventional recreational drug use in pregnancy. Semin Fetal Neonatal Med 2019;24:142–148. 30744980 10.1016/j.siny.2019.01.010

[45] Anghelescu DL Tesney JM . Neuropathic pain in pediatric oncology: a clinical decision algorithm. Paediatr Drugs 2019;21:59–70. 30761460 10.1007/s40272-018-00324-4 PMC6779139

[46] Abdallah CG Averill LA Krystal JH . Ketamine as a promising prototype for a new generation of rapid-acting antidepressants. Ann N Y Acad Sci 2015;1344:66–77. 25727103 10.1111/nyas.12718 PMC4412785

[47] Komatsu R You J Mascha EJ . Anesthetic induction with etomidate, rather than propofol, is associated with increased 30-day mortality and cardiovascular morbidity after noncardiac surgery. Anesth Analg 2013;117:1329–1337. 24257383 10.1213/ANE.0b013e318299a516

[48] Baradari AG Alipour A Habibi MR . A randomized clinical trial comparing hemodynamic responses to ketamine-propofol combination (ketofol) versus etomidate during anesthesia induction in patients with left ventricular dysfunction undergoing coronary artery bypass graft surgery. Arch Med Sci 2017;13:1102–1110. 28883852 10.5114/aoms.2016.63193 PMC5575215

[49] Habibi MR Baradari AG Soleimani A . Hemodynamic responses to etomidate versus ketamine-thiopental sodium combination for anesthetic induction in coronary artery bypass graft surgery patients with low ejection fraction: a double-blind, randomized, clinical trial. J Clin Diagn Res 2014;8:Gc01–Gc05. 10.7860/JCDR/2014/10237.5006 PMC4253182 25478364

[50] Kaushal RP Vatal A Pathak R . Effect of etomidate and propofol induction on hemodynamic and endocrine response in patients undergoing coronary artery bypass grafting/mitral valve and aortic valve replacement surgery on cardiopulmonary bypass. Ann Card Anaesth 2015;18:172–178. 25849685 10.4103/0971-9784.154470 PMC4881645

[51] Yao YT He LX Fang NX . Anesthetic induction with etomidate in cardiac surgical patients: a PRISMA-compliant systematic review and meta-analysis. J Cardiothorac Vasc Anesth 2021;35:1073–1085. 33384231 10.1053/j.jvca.2020.11.068

[52] Zed PJ Abu-Laban RB Harrison DW . Intubating conditions and hemodynamic effects of etomidate for rapid sequence intubation in the emergency department: an observational cohort study. Acad Emerg Med 2006;13:378–383. 16531603 10.1197/j.aem.2005.11.076

[53] Schraag S Pradelli L Alsaleh AJO . Propofol vs. inhalational agents to maintain general anaesthesia in ambulatory and in-patient surgery: a systematic review and meta-analysis. BMC Anesthesiology 2018;18:162. 30409186 10.1186/s12871-018-0632-3 PMC6225663

[54] Miller AL Theodore D Widrich J . Inhalational Anesthetic. StatPearls. StatPearls Publishing; 2022. 32119427

[55] Zuo Z . Are volatile anesthetics neuroprotective or neurotoxic? Med Gas Res 2012;2:10. 22510328 10.1186/2045-9912-2-10 PMC3353836

[56] Bauer AM Lovich-Sapola JA Lovich-Sapola JA . Inhalational Anesthetics. Anesthesia Oral Board Review: Knocking Out the Boards. Cambridge University Press; 2009:45–46.

[57] Wang J Cheng CS Lu Y . Volatile anesthetics regulate anti-cancer relevant signaling. Front Oncol 2021;11:610514. 33718164 10.3389/fonc.2021.610514 PMC7952859

[58] Ackerman RS Luddy KA Icard BE . The effects of anesthetics and perioperative medications on immune function: a narrative review. Anesth Analg 2021;133:676–689. 34100781 10.1213/ANE.0000000000005607

[59] Chen RM Wu CH Chang HC . Propofol suppresses macrophage functions and modulates mitochondrial membrane potential and cellular adenosine triphosphate synthesis. Anesthesiology 2003;98:1178–1185. 12717140 10.1097/00000542-200305000-00021

[60] Melamed R Bar-Yosef S Shakhar G . Suppression of natural killer cell activity and promotion of tumor metastasis by ketamine, thiopental, and halothane, but not by propofol: mediating mechanisms and prophylactic measures. Anesth Analg 2003;97:1331–1339. 14570648 10.1213/01.ANE.0000082995.44040.07

[61] Tazawa K Koutsogiannaki S Chamberlain M . The effect of different anesthetics on tumor cytotoxicity by natural killer cells. Toxicol Lett 2017;266:23–31. 27940100 10.1016/j.toxlet.2016.12.007 PMC5209272

[62] Krumholz W Abdulle O Knecht J . Effects of i.v. anaesthetic agents on the chemotaxis of eosinophils in vitro. Br J Anaesth 1999;83:333–335. 10618953 10.1093/bja/83.2.333

[63] Zhang Y Li RM Wang C . Etomidate inhibits nuclear factor-κB through decreased expression of glucocorticoid receptor in septic rats. Mol Med Rep 2016;14:5760–5766. 27878281 10.3892/mmr.2016.5947

[64] Devlin EG Clarke RS Mirakhur RK . Effect of four i.v. induction agents on T-lymphocyte proliferations to PHA in vitro. Br J Anaesth 1994;73:315–317. 7946855 10.1093/bja/73.3.315

[65] Liu FL Chen TL Chen RM . Mechanisms of ketamine-induced immunosuppression. Acta Anaesthesiol Taiwan 2012;50:172–177. 23385040 10.1016/j.aat.2012.12.001

[66] Nishina K Akamatsu H Mikawa K . The effects of clonidine and dexmedetomidine on human neutrophil functions. Anesth Analg 1999;88:452–458. 9972773 10.1097/00000539-199902000-00042

[67] Franks NP . General anaesthesia: from molecular targets to neuronal pathways of sleep and arousal. Nat Rev Neurosci 2008;9:370–386. 18425091 10.1038/nrn2372

[68] Yuki K Hou L Shibamura-Fujiogi M . Mechanistic consideration of the effect of perioperative volatile anesthetics on phagocytes. Clin Immunol 2021;222:108635. 33217544 10.1016/j.clim.2020.108635 PMC7856197

[69] Tecchio C Micheletti A Cassatella MA . Neutrophil-derived cytokines: facts beyond expression. Front Immunol 2014;5:508. 25374568 10.3389/fimmu.2014.00508 PMC4204637

[70] Stollings LM Jia LJ Tang P . Immune modulation by volatile anesthetics. Anesthesiology 2016;125:399–411. 27286478 10.1097/ALN.0000000000001195 PMC5074538

[71] Koutsogiannaki S Schaefers MM Okuno T . From the cover: prolonged exposure to volatile anesthetic isoflurane worsens the outcome of polymicrobial abdominal sepsis. Toxicol Sci 2017;156:402–411. 28003439 10.1093/toxsci/kfw261 PMC5412087

[72] Kotani N Hashimoto H Sessler DI . Intraoperative modulation of alveolar macrophage function during isoflurane and propofol anesthesia. Anesthesiology 1998;89:1125–1132. 9822000 10.1097/00000542-199811000-00012

[73] Plein LM Rittner HL . Opioids and the immune system—friend or foe. Br J Pharmacol 2018;175:2717–2725. 28213891 10.1111/bph.13750 PMC6016673

[74] Eisenstein TK . The role of opioid receptors in immune system function. Front Immunol 2019;10:2904. 31921165 10.3389/fimmu.2019.02904 PMC6934131

[75] Garcia JB Cardoso MG Dos-Santos MC . Opioids and the immune system: clinical relevance. Rev Bras Anestesiol 2012;62:709–718. 22999403 10.1016/S0034-7094(12)70169-1

